# Role of hepatitis c virus in hepatocellular carcinoma and neurological disorders: an overview

**DOI:** 10.3389/fonc.2022.913231

**Published:** 2022-07-29

**Authors:** Mohd Suhail, Sayed Sartaj Sohrab, Mohammad Amjad Kamal, Esam Ibraheem Azhar

**Affiliations:** ^1^ King Fahd Medical Research Center, King Abdulaziz University, Jeddah, Saudi Arabia; ^2^ Department of Medical Laboratory Sciences, Faculty of Applied Medical Sciences, King Abdulaziz University, Jeddah, Saudi Arabia; ^3^ Special Infectious Agents Unit, King Fahd Medical Research Center, King Abdulaziz University, Jeddah, Saudi Arabia; ^4^ West China School of Nursing/Institutes for Systems Genetics, Frontiers Science Center for Disease-Related Molecular Network, West China Hospital, Sichuan University, Chengdu, China; ^5^ Enzymoics Novel Global Community Educational Foundation, Hebersham, NSW, Australia

**Keywords:** hepatitis C virus (HCV), hepatocellular carcinoma (HCC), HCV diagnosis, treatment, central nervous system (CNS), neurological disorders

## Abstract

The hepatitis C virus (HCV) causes serious issues, affecting 71 million people globally. The most common manifestations range from chronic hepatitis to liver cirrhosis, leading to hepatocellular carcinoma. Many mechanisms are known to play an important role in HCV-induced HCC. The interaction of viral proteins with host cells results in oxidative stress damage, liver inflammation, and irregularities in signaling pathways. These results in the activation of oncogenes and metabolic disturbances, liver fibrosis, and angiogenesis. Additionally, some non-coding RNAs (ncRNAs) and toll-like receptors have been identified and play a significant role in HCC development. This virus is also associated with impairment of the central nervous system, resulting in acute or sub-acute encephalopathy and inflammatory disorders. Neurological disorders are associated with the inflammatory responses of many cells, including microglia and astrocytes. Additionally, there are many other extrahepatic manifestations, including neurological disorders such as depression and fatigue, in 50% of infected patients. These manifestations include neuro-invasion, immune-mediated damage, neurotransmitter alterations, sensory-motor polyneuropathy, sensitivity loss, weakness of the leg, and cryoglobulinemia, which significantly results in a reduced quality of life. HCV infection may be improved using an appropriate diagnosis and direct antiviral therapy for sustained virological response. However, the success of therapy depends on the symptoms and organ damage, diagnosis, and therapeutic strategies applied. Some published reports have discussed that HCV is associated with both HCC and neurological disorders. Additionally, it has also been observed that individuals with HCC also develop neurological disorders compared with individuals with HCV alone. This review aims to provide an overview of the latest information about the relationship between HCV-induced HCC and their role in neurological disorders. Additionally, we have also discussed the progress made in the diagnosis, physio-pathological mechanisms, and strong antiviral therapies developed for HCV infection and HCC, as well as the latest advancements made in the study of the neurological disorders associated with HCV infection.

## Introduction

Hepatitis C virus (HCV) is an RNA virus responsible for liver inflammation and the development of hepatocellular carcinoma (HCC). HCV has six main genotypes and four of them are the most common in low-income countries, while genotype 1 is the most common in middle- and high-income countries (1). The virus causes both acute and chronic hepatitis, including liver cirrhosis and the development of cancer. HCV is a blood-borne virus and is commonly transmitted through the unsafe use of medical equipment, especially syringes, and non-tested blood or blood products or sexual practices that lead to blood. HCV can also be passed from mother to newborn. The HCV requires 2 weeks to 6 months as an incubation period for symptom development. The most common symptoms include fever, fatigue, decreased appetite, nausea, vomiting, abdominal pain, dark urine, pale feces, joint pain, and jaundice. But 80% of infected individuals do not exhibit symptoms in the early stages of infection. The diagnosis of HCV infection is currently being performed by both serological and molecular assays. Early diagnosis can prevent the transmission and spread of the virus. Recent evidence shows that HCV infection is increasing at an alarming rate and varies widely across the global population. The total global infection of HCV is estimated at 177.5 million infected adults, and it is expected that 1.6 million new infections occur every year (2, 3). Currently, no vaccines are available against HCV, but antiviral therapy and direct-acting antivirals (DAAs) are being used to cure HCV-infected patients. The highest prevalence of HCV has been observed in many countries, including Cameroon, Egypt, Gabon, Georgia, Mongolia, Nigeria, and Uzbekistan, with a history of “iatrogenic” infections, which is a major risk factor (1).

## Viral characteristics, pathogenesis, and clinical manifestations

The HCV belongs to the *Flaviviridae* with an almost 9.6 kb RNA genome encoding a single open reading frame (ORF). This ORF results in the production of an approximately 3,000-amino-acid-long polyprotein (4). The polyprotein is subsequently translated and processed into three structural (core (C), envelope 1 (E1), and envelope 2 (E2) and seven non-structural proteins (viroporin p7, non-structural proteins 2 (NS2), NS3, NS4A, NS4B, NS5A, and NS5B). The non-structural proteins have critical functions in viral replication. Several viral proteins also appear to play an important role in the evasion of host immune responses. In addition to infecting hepatocytes, HCV also infects dendritic cells (DCs), B cells, and peripheral blood mononuclear cells (PBMC) (5). The HCV viral genome is uncoated and releases positive sense RNA into the hepatocyte cytosol where it replicates in the major cellular system and performs protein synthesis (6).

Extra hepatic manifestations (EHMs) are associated with HCV. The proliferation of B lymphocytes occurs after virus infection, and rheumatoid factors or cryoglobulins are associated with HCV infection. HCV is associated with mainly two types of disease progression, which are known as acute and chronic hepatitis C viral infections. Individuals with acute HCV infections are primarily asymptomatic and suffer liver damage. But more than 70% of acute HCV-infected individuals develop chronic HCV. The viral RNA can be detected in the blood of acute patients even after six months. Approximately 30% of the infected individuals undergo spontaneous clearance of the virus infection after six months. The risk factors for chronic HCV depend on gender, ethnicity, co-infection with HIV, and the immunocompromised state of the individuals. The extrahepatic manifestations of chronic patients who develop mixed cryoglobulinemia, membranoproliferative glomerulonephritis, lichen planus, vitiligo, keratoconjunctivitis sicca, and lymphoma. HCV infection results in an elevated level of ALT that further works as an indicator of disease progression. It is well known that, around 80% of HCV-infected patients develop chronic infection, which ultimately causes a more severe form of liver problem such as liver cirrhosis and HCC (5). It has been reported that more than 90% of HCV-induced liver cancer is due to the dysregulation of a complex epigenetic network (7). HCV causes long-term liver inflammation that indicates the advanced stages of liver diseases, including fibrosis, cirrhosis, and hepatocellular carcinoma (HCC), which finally leads to the death of infected patients. It is estimated that 1.5 million individuals are infected with HCV every year. However, only 20%–30% develop liver cirrhosis, of which 1%–4% develop HCC (8).

## Hepatitis c virus induces hepatocellular carcinoma (hcc)–host interaction

A basic understanding of the molecular mechanisms involved in hepatocarcinogenesis is essential for developing possible therapeutic approaches. The development of HCV-induced HCC is a slow-growing process that takes 20–40 years in infected patients (9). Several studies suggest that liver damage is a complex process caused by chronic HCV infection (4, 10). It is well known that hepatocytes are the primary host for HCV infection. Additionally, some studies have suggested that dendritic cells, B cells, and T cells in peripheral blood mononuclear cells (PBMC) are additional reservoirs for HCV infection (11, 12). Various animal model studies suggest that different viral proteins may be directly involved in the pathogenesis of hepatocellular carcinoma (9, 13–16). In a recent study, it was observed that there is a significant increase in triglycerides, cholesterol, low-density lipoprotein, and cholesterol in patients with CHC. The higher value is associated with a higher risk of cirrhosis development (17).

**Figure 1:**
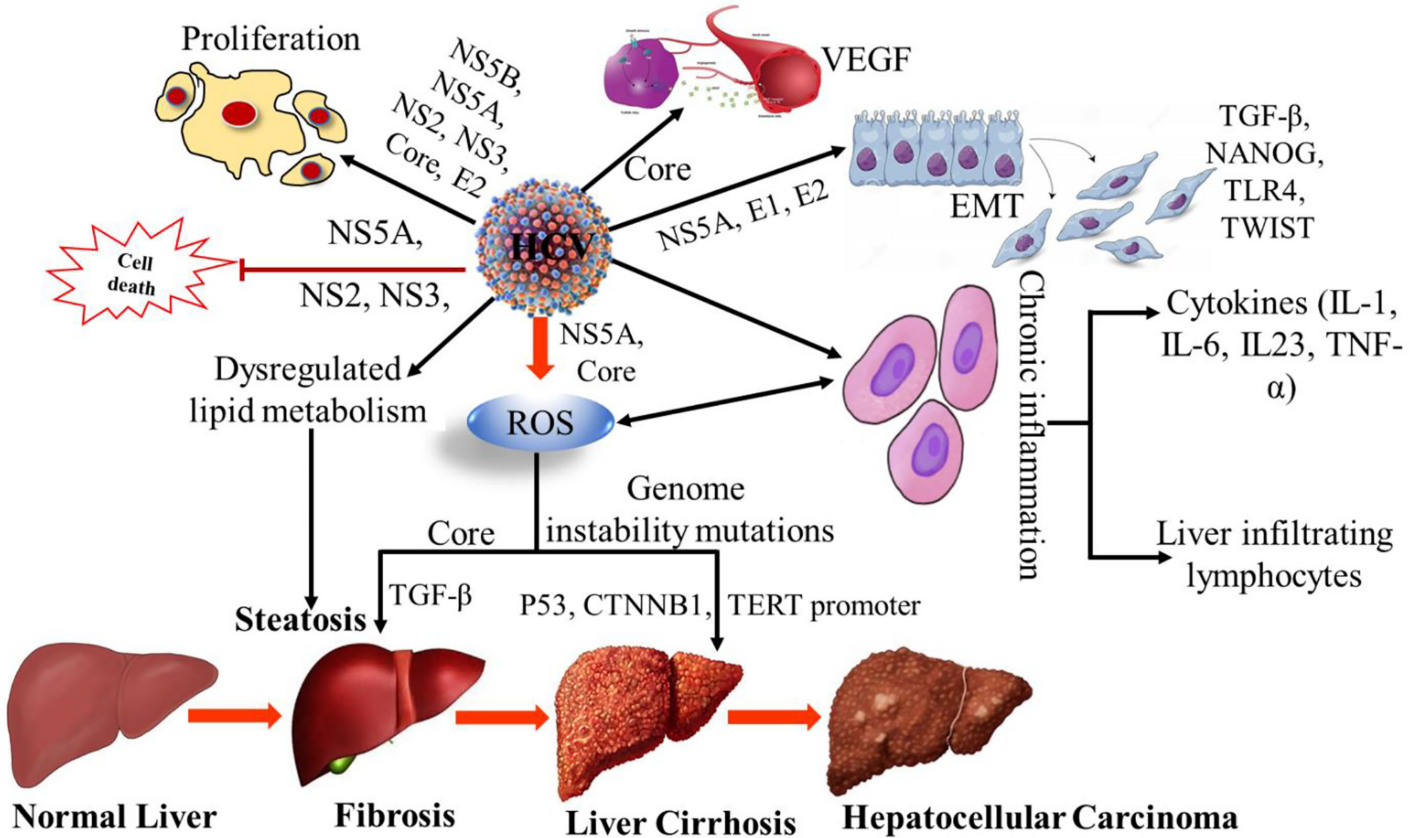
Mechanism of HCV induced hepatocellular carcinoma. VEGF, Vascular endothelial growth factor; EMT, Epithelial–mesenchymal transition; TGF-β, Transforming Growth Factor-β; TLR4, Toll-like receptor 4; TWIST, Twist-related protein; ROS, Reactive oxygen species; CTNNB1, Catenin Beta 1; TERT, Human telomerase reverse transcriptase; TNF-α, Tumor necrosis factor alpha; NS, non-structural; IL-1; Interleukin-1, IL-6; Interleukin 6, IL-23, Interleukin-23.

HCV-NS3 is a serine protease (4) and it plays an important role in the neoplastic transformation process by inducing the acquisition of the hepatocyte clones in a proliferative condition. In addition, escape from the host cell surveillance mechanisms (18). In a study, it has been suggested that other HCV proteins, such as core and envelope (E2), stimulate cell growth and heteroplastic degeneration (19, 20). The HCV core protein appears to be involved majorly in the pathogenesis of HCC. HCV core proteins have high oncogenic potential as they alter the intracellular signaling cascade of the protein kinase and cause oxidative stress (21). Specifically, HCV core protein can also induce the overproduction of reactive oxygen species (ROS). In addition, it increases lipid peroxidation and disrupts mitochondrial function by altering the double-layer lipoprotein of the mitochondrial membrane (22, 23). HCV core protein-induced ROS damages the host cell and accumulates the genetic variation, leading to cancer (23, 24). In addition, it inhibits DNA repair mechanisms that are impaired by oxidative stress and modifies different intracellular antioxidant systems (25, 26). Core protein also has the properties to inhibit the tumor suppressor genes Tp53, TP73, RB transcriptional corepressor 1 (RB1), and cyclin-dependent kinase inhibitor 1A (CDKN1A) (27). HCV infection alters the transforming growth factor-beta (TGF-β) signaling pathway (**Figure 1**), resulting in the progression of liver injury and an increase in the risk of HCC (28). TGF-β has antiproliferative and proapoptotic properties. In addition, it plays a role in epithelial-mesenchymal transition (EMT) and has been shown to have protumoral activities. EMT is a process in which epithelial degeneration, fibrogenesis, metastasis, and invasiveness occur (29). Regarding the dual activity of TGF-β in cancer, it was reported that during chronic inflammation in HCV patients, the tumor-suppressor activity of TGF-β shifts into fibrogenic and thus leads to the risk of HCC (30, 31). Evidence suggested that different inflammatory cytokines such as IL-1, IL-23, IL-6, and lymphotoxins (LT) were involved in the development and progress of liver inflammation and HCC (32). Additionally, it appears that HCV NS5A stimulates the PI3K-Akt survival pathway through down-regulation of the phosphatase and tensin homolog (PTEN) to relieve its inhibitory effect on the PI3K-Akt signaling pathway and thus may indirectly inhibit apoptosis (33, 34). Recently, a large cohort study in the United States revealed that HCV genotype 3 infections had an 80% higher risk for HCC development as compared to genotype 1. While the Southeast Asian cohort study showed that HCV genotype 6 is strongly associated with HCC development. The sustained virological response (SVR) was observed in more than 90% of the treated individuals and showed a reduction of HCC risk against all the genotypes (35, 36).

## Molecular mechanism of hcv induced hcc

Viral proteins play an important role in chronic infection. They show the immunosuppressive activity of dendritic cells (DC), natural killer (NK) cells, and T cells. This activity led to the strong establishment of a chronic infection. During the infection, HCV uses several mechanisms that lead to the development of HCC (**Table 1**). These viral proteins induce intracellular oxidative stress damage, persistent liver inflammation, and deregulation of signaling pathways. HCV causes fibrosis and HCC by metabolic reprogramming. During chronic infection, the immune responses are significantly impaired. Many mechanisms that have been proposed for the failure of the host response to viral clearances. These mechanisms are known as; variations in the viral genome, suppression of immunogenic response by viral proteins, inhibition of innate immune responses, dysfunction of T lymphocytes and the involvement of regulatory T cells (5, 36, 40).

**Table 1:** Association of HCV in the development of Hepatocellular Carcinoma (HCC).

S. No.	HCV (Viral Properties)	Descriptions	References
1	Viral Proteins	The viral polyprotein is such as core (C), envelope 1 (E1), and envelope 2 (E2) and non-structural proteins (NS2), NS3, NS4A, NS4B, NS5A, and NS5B) interacts with host proteins and results into HCC.	(4)
2	Pathogenesis	Acute and chronic hepatitis C viral infections. More than 70% of the acute HCV-infected individuals develop chronic HCV.	(1)
3	Clinical manifestations	fever, fatigue, decreased appetite, nausea, vomiting, abdominal pain, dark urine, pale feces, joint pain, and jaundice.	(7)
4	Liver fibrosis and Angiogenesis	HCC includes liver inflammation, fibrosis, and Angiogenesis.	(9)
5	Molecular mechanisms	Disturbances in the signaling pathways and induction of oncogenes which leads to HCC.	(5)
6	Signaling and host Immune Response	Oxidative stress and oncogenic and toll like receptors.	(7)
7	ncRNA	The non-coding RNA play an important role in HCC development.	(3)
8	Diagnosis	These include serological and molecular assay.	(37)
9	Vaccine	An effective and affordable vaccines is urgently required.	(3)
10	Potential biomarkers	Some potential biomarkers have been identified to detect the HCC.	(28)
11	DAAs	The role of direct acting antivirals has been discussed in the text.	(38)
12	Scientific Evidence	Currently, significant evidence has been published related to HCV induced HCC.	(39)

### Host mmune response to hepatitis c virus infection

During the early phase of acute infection, a high level of ISGs is exhibited with a high viral load, reflecting the reduction in viral eradication. The NK cells also show important antiviral and immunoregulatory roles during acute HCV infection. These cells also showed elevated levels of IFNγ production and high cytolytic activity in HCV patients. It has also been observed in unintentionally infected healthcare professionals with HCV that the NK cells get activated, but no symptoms of acute infection were observed. The expression of natural killer group 2, member A (NKG2A), blunted the activation of dendritic cells on the surface of NK cells infected with HCV (1).

In the host adaptive immune response to acute HCV infection, the activation of response is observed several weeks after the HCV infection. This delay happens due to the activation of early activation of innate immune responses. A study conducted on chimpanzees with HCV that, antigens resulted in the activation of peripheral and intrahepatic CD4+ T-cell responses. Interestingly, the production of IFNγ was also observed by both peripheral and intrahepatic CD8+ T cells in either partially or fully HCV viral-eradicating chimpanzees.

In the host adaptive immune response to chronic hepatitis C infection, the viral persistence is linked to the low response of T cells as well as multi-specific CD4+ and CD8+ T-cell responses, which is related to viral clearance. The virus-specific CD8+ T cells were characterized by the low proliferation and reduced secretion of IFNγ in chronically infected HCV patients (41). The downregulation of TIM3 and upregulation of LAG3 on the surface of their CD8+T cells have been observed in HCV patients with sustained virological responses (SVR). The virus-specific CCR7-CD8+ T-reg cell population was identified for the first time in chronic HCV patients. HCV bypasses humoral immunity through many alternative pathways. The viral persistence and emergence of host humoral immunity occur due to the presence of viral quasi-species, cell-to-cell transmission of HCV, the interaction of HCV-GAGs with HDL and SCARB1 receptors, and distinct glycans on E2 proteins (1).

In the innate immune response to chronic hepatitis C infection of the host, viral loss at the NK cell surface is associated with HCV persistence. The NK cells of chronically infected patients lack NKp46 and NKp30 receptors compared with controls because of the low expression of natural cytotoxicity receptors. But these NK cells have a high level of NKG2A inhibitory molecule, which further predisposes them to viral chronicity. The phosphorylation and activation of STAT1 were observed in NK cells of chronically infected HCV patients. In an *in vitro* study, it was observed that, when directly co-cultured in HCV-infected hepatocytes, the low expression of NKG2D, NKp30, and IFNγ on NK cells happened to their reduced cytolytic activity. The macrophages engulf HCV particles, resulting in the high release of IL6 and IL-1β into the surrounding microenvironment. HCV uptake triggers the apoptosis of macrophages. The release of CCL5 by macrophages was also observed after co-culture with HCV-infected hepatocytes. They further activate the high-level expression of fibrogenic and inflammatory markers, which leads to liver fibrosis (1, 42).

### Epigenetic mechanisms and hcv-induced hepatocellular carcinoma (hcc)

Despite advanced research and progress toward HCV-induced HCC, the mechanism of carcinogenesis has not been fully understood. It involves a complex mixture of epigenetic regulation and signaling pathways. Recently, 18 specific gene targets and different signaling pathways have been identified. These include histone modification, DNA methylation, and dysregulation of gene expression, which lead to HCC development (7). The results of DNA methylation, which includes DNA hypermethylation and hypomethylation, are significantly associated with the development of cancer. In a study, it was reported that the hypermethylation of a tumor suppressor gene at the promoter region significantly contributes to the carcinogenic processes of HCV-induced HCC. The RNA sequence analysis showed the low expression of proteins due to hypermethylation of tumor-suppressive genes, whereas the hypomethylation of oncogenic genes results in the high expression of proteins. The properties of the gene regulatory network in HCV-induced HCC were investigated, and only 14 genes were involved in secreted frizzled-related protein 1 (SFRP1) as well as another cellular process. The high expression level of SFRP1 has a meaningful impact on the survival rates of HCC patients. HCV infection promotes epigenetic inactivation of SFRP1 and leads to HCC. A total of 11 tumor suppressor genes have been identified which are directly involved in HCV-induced HCC development. These are known as RASAL1, EGLN3, CSMD1, CDKN2A, BCORL1, SFRP1, ZNF382, RUNX3, LOX, RB1, and P73.

Histone modification plays an important role in HCC development and has been intensively studied in various cancer models. The modifications include histone methylation, acetylation, phosphorylation, sumoylation, and ubiquitination. In the case of HCV-induced HCC, the overexpression of KDM5B/JARID1B, a member of the JmjC histone demethylase, results in HCC cell proliferation by regulating downstream E2F1 and E2F2 genes (7, 43). The regulation of histone acetylation is controlled by histone acetyltransferases (HATs) and histone deacetylases (HDACs) that lead to HCC development in HCV-infected patients. HCC-related histone acetylation usually involves multiple biological processes. For example, iron overload is a risk factor for HCV to HCC progression in the liver. Many histone acetylation have been reported, such as histone H3 acetylated on lysine 9 (H3K9Ac), histone 3 acetylated on lysine 27 (H3K27Ac), H2A acetylated on lysine 5 (H2AK5ac), and H3 acetylated on lysine 14 (H3K14Ac) (7, 44).

## Signaling pathways in hcv-induced hcc

Based on the current reports, it is evident that the signaling pathway and molecular mechanism of HCV-induced HCC are very complex. Several studies have shown that the proteins encoded by HCV-RNA can be significantly modulated by many signaling pathways, including the Wnt signaling pathway, Ras/MAPK signaling pathway, p53 signaling pathway, and JAK-STAT and PI3K-AKT pathways. Furthermore, these pathways are tightly linked with many cellular functions, including regulation, proliferation, and apoptosis (7).

### HCV-induced oxidative stress signaling

HCV-infected cells produce reactive oxygen species (ROS). The HCV core proteins (E1/E2 and NS4B) activate oxidative stress using calcium efflux by inducing ER stress and UPR. The HCV-core protein interacts with the outer membrane of mitochondria and heat shock protein Hsp60 and triggers the Ca2+ from the ER and accumulation in the mitochondria, leading to the expression of oxidoreductin 1α (ERO1α) in the ER. This accumulation promotes ROS production by changing the respiratory chain (45). Additionally, HCV-NS5A enhances the expression of cytochrome P4502E1 (CYP2E1) and NADPH oxidase 1 and 4 (NOX1 and 4), resulting in elevated ROS, including superoxide and hydrogen peroxide, and downregulates SOD1 and SOD2 induces catalase activity. NS4B and NS5A activate NF-κB and STAT3 expression, disrupt growth factor-beta 1 (TGF-β1) secretion and the calcium homeostasis, and finally lead to progression of liver fibrosis. It has been revealed that HCV activates the Nrf2/ARE axis, which leads to ROS scavenging and prevention of ROS accumulation, and finally protects them from the antiviral/lethal effects of the infected cells (46).

### HCV-induced oncogenic signaling

During the progression of liver disease to HCC, many common mutations occur, resulting in the conversion of normal cells to tumorigenic cells. Based on single-cell and next-generation sequencing, it was observed that these cells are highly heterogeneous within the same HCV-infected individual. The trsults of the sequence analysis showed that many genes are strongly associated with the progression of HCC. These genes are known as TERT, TP53, CTNNB1, Wnt/β-catenin signaling protein AXIN1, ARID1A and ARID2, NFE2L2, and KEAP1, RAS/MAPK signaling (RPS6KA3), and the JAK/STAT signaling cascade activator (KAK1) genes (36). The virus-induced signaling pathways promote viral persistence by diverting the antiviral responses induced by host cells to control apoptosis and ensure the survival of infected cells. Additionally, these signals also play an important role in the regenerative process during liver injury and maintain the balance between the pro-inflammatory and proliferative signals (46, 47). HCV maintains a strategy for EGFR signaling by retaining EGFR and altering the expression of other ErbB receptors in the early endosome *via* NS5A that leads to the accumulation of more EGFR in the infected cells. HCV-NS5A promotes virus replication by associating with Raf-1 kinase. This suggests direct virus-host dependency and pathway-associated transcriptional changes in HCV-infected patients (46–48).

### TLR response to hcv infection

Toll-like receptors (TLRs) are the most important molecules that produce cytokines and signaling pathways that provide a link between innate and acquired immunity. Currently, different types of TLR have been reported that interact with HCV proteins and nucleic acids in HCV-infected cells. TLR3 senses the HCV through the detection of dsRNA intermediates in infected hepatoma cells and activates the TLR3-signaling cascade, resulting in the production of type I and II IFNs and limiting the virus replication. TLR2 and TLR4 are known for the modulation of proinflammatory responses by HCV proteins, and they have been detected in Raji cells and peripheral blood mononuclear cells (PMBCs). An association between TLR4 gene polymorphisms and chronic HCV infection has also been reported in a Saudi Arabian population, which suggests the significant role of TLR4 during HCV infection and HCC development (49–51).

### Inflammation of the liver, fibrosis, and angiogenesis

Currently, many factors are known to contribute significantly to inflammation and liver fibrosis. These factors include viral particles, host-pathogen-associated molecular patterns, and damage-associated patterns. These patterns activate macrophages, which leads to induction and hepatic inflammation. Based on the biomarkers or aminotransferase levels, it has been observed that the activation of macrophages has diminished after the antiviral therapy of chronic hepatitis patients. HCV-induced fibrosis has been observed more in HIV-coinfected patients than in mono-infected patients (52, 53).

Angiogenesis is a physiological process that is closely linked to HCC disease progression. The generation of new blood vessels occurs during angiogenesis as well as at the advanced stage of liver disease. The process of angiogenesis is activated when the tumorigenic tissue needs more oxygen and nutrient supply. The HCC cells secrete the factors responsible for the activation of endothelial cells by VEGF and FGF. The expression levels of the angiogenic growth factors VEGF-A, angiopoietin-2, and PDGF are elevated in HCC tissues. Additionally, the activation of VEGF may occur by many other cellular pathways, which include PI3K/Akt, ERK1/2, NF-κB, and STAT3, which stabilize HIF-1α (28).

## Association of non-coding RNAs in HCV-induced HCC

Various types of non-coding RNAs (ncRNAs), including miRNAs and long noncoding RNAs (lncRNAs), have been identified in high-throughput transcriptome studies in eukaryotes. These ncRNAs play an important role in various cellular processes, including the HCC (54). As discussed, 18 genes are important in HCC and these genes are tightly associated with ncRNAs. These ncRNAs regulate the cellular process by upregulating or downregulating the functions of various genes in HCV-induced HCC. But still, the mechanism needs to be further explored (7). During the virus–host interaction of the HCV-infected HCC, the miRNAs exert an antiviral effect on HCV replication and infection. Currently, more than 1,000 miRNAs have been identified and miR-122 is one of the most abundant in the liver, which is strongly associated with HCV-induced HCC. Currently, a few miRNAs (miRNA-484, 524, 615, and 628) have been identified in HCC patients, and they are being used as potential biomarkers for liver cirrhosis. In HCV-induced HCC, there are only 2 lncRNAs reported and they are known as HIF and PAR5. Additionally, some lncRNAs (LINC01419, BC014579, AK021443, RP11-401P9.4, RP11-304 L19.5, CTB-167B5.2, and AF070632) are also known to be differentially expressed in HCC tissues and they are being used as biomarkers for HCC detection (55).

## HCV diagnosis

The diagnosis of HCV infection is currently being performed using many assays, including serological, molecular, and rapid detection tests and biomarkers. Based on the WHO recommendation, the diagnosis of HCV should be performed by detecting the anti-HCV antibodies by serological assay (antibody or antibody/antigen) using either an RDT or a laboratory-based immunoassay. Additionally, the HCV RNA can be easily detected by a molecular assay based on nucleic acid-detection methods such as automated real-time PCR (37, 56). The significant use and quality of rapid detection tests mainly depend on the accuracy, specificity, and sensitivity as well as the positive and negative ratio, which may vary with disease prevalence in different populations. Currently, many kits such as GeneXpert Omni (Cepheid, Sunnyvale, CA) and small devices such as Genedrive^®^ (Genedrive Diagnostics, Manchester, UK) have been developed for HCV–RNA detection. Soon, smartphone-based applications, biosensors, lab-on-a-chip NAT-based amplification, and wearable devices will offer new strategies for HCV diagnosis (37, 57–59).

### Potential biomarkers for HCC detection

Biomarkers play an important role in the detection of HCV-induced HCC. Currently, many targets can be used for developing serum-based novel biomarkers. These markers can accurately measure the virus-induced cellular stress that leads to HCC. To date, only two serum fibrosis scores, known as fibrosis 4 (FIB-4) and the AST to platelet ratio index (APRI), are being used. Currently, only one HCC tumor biomarker, AFP (70-kDa-serum glycoprotein), is being used for HCC detection. The serum levels of AFP are increased during HCC. The AFP is present in three different forms, known as AFP-L1, AFP-L2, and AFP-L3. The level of AFP-L1 is elevated during chronic hepatitis and liver cirrhosis, while the level of AFP-L3 increases during HCC development (28).

## DAAs treatment and HCC

Direct-acting antivirals (DAAs) therapy has vastly improved SVR rates in HCV-induced HCC patients. Many studies have been conducted to assess the benefits and harms of DAAs in chronic HCV patients. However,information about the beneficial effects of DAA therapy is still limited. A non-invasive method known as vibration-controlled transient elastography (VCTE) with a controlled attenuation parameter (CAP), 12 weeks after SVR, has been used in the last few years for hepatic steatosis and fibrosis detection. A significant increase was observed in hepatic steatosis and a reduction in fibrosis score after the eradication of the virus by DAAs. So, a long-term follow-up must identify the hepatic steatosis post-SVR and the risk of more severity in HCV-infected patients (38, 60). A recent study was conducted on 51 different DAAs by including 138 trials with a total of 25,232 participants. Eighty-four trials involved DAAs on the market or under development. Fifty-seven trials administered DAAs that were discontinued or withdrawn from the market. Based on the results, it was finally concluded that the rate of hepatitis C morbidity and mortality was relatively low and did not influence the serious adverse events. The data were not enough to judge the harmful and beneficial effects of DAAs on chronic HCV. The quality of evidence for the DAAs was very low or low quality because of many limitations, which include lack of blinding, lack of relevant data, missing data, no published protocol, etc. (61). Recently, the effectiveness of direct-acting antiviral agents (DAAs) for HCV-infected patients in the Kingdom of Saudi Arabia (KSA) has been evaluated. It was observed that an overall HCV cure rate of 97% following treatment with DDA, was prescribed in the private sector (28, 62).

### Development of HCV vaccine

Despite DAA-based treatments, the rate of HCV infection is increasing at an alarming rate globally. The risk of re-infection after DAA treatment is a major challenge. There is an urgent need to develop a protective and affordable vaccine against HCV. The major challenge in a developing highly effective vaccine is due to the complex nature of the virus, which includes genetic diversity, seven genotypes, sub-types, viral variants, and antibody responses. Currently, only two vaccine candidates have been designed using different strategies and platforms. They have reached an advanced level of clinical trials. The first vaccine contains the viral envelope glycoproteins called E1/E2 mixed with MF59 adjuvant, and the second is based on the use of recombinant replication-defective adenovirus with whole non-structural proteins. The initial data for the first vaccine suggested strong neutralizing antibody responses in chimpanzees and neutralizing antibody responses as well as a CD4 T-cell response in human volunteers. For the second vaccine, the human volunteers show a broad and strong HCV-specific T-cell response (28, 63, 64).

## Viral pathogenesis and neurological disorders

There are many mechanisms known and hypothesized for the role of HCV in the occurrence and development of neurological and psychiatric symptoms. The association of HCV in the development of neurological disorders has been presented in **Table 2**. This includes neuro-invasion and direct damage to the central nervous system (CNS). It has been reported that the virus enters the major cells and replicates easily in the infected cells. The presence of HCV-RNA in the CNS and cerebrospinal fluid of patients has been sequenced and reported in many published papers (3, 6, 69), but the level of viral RNA was observed to be 1,000–10,000 times lower in those cells as compared to liver cells (69). Many EHMs, including neurological disorders, are rheumatologic/immunologic (75, 76). There are many CNS complications such as cerebrovascular, neuropathology, encephalic inflammation, autoimmune, meningeal, and rheumatic disorders, which are known with the HCV infection (69). The brain dysfunction is caused by chronic HCV infection in those patients without cirrhosis or cryoglobulinemia (3, 77). Based on a recent study, it has been observed that neurological and psychiatric symptoms develop in patients with non-cirrhosis symptoms without significant correlation with the virus replication rate and liver disease severity (6). So, during the diagnosis of a patient with neurological disorders, it should be considered that HCV could be an important etiological factor (6). A study suggested that more than 50% of HCV-infected patients are associated with neurological and neuropsychiatric problems (6). Additionally, the viral quasi-species was detected in the lymphoid tissues and peripheral blood mononuclear cells (PBMC), which indicates that the virus can enter the CNS through the trojan horse mechanism after infecting the PBMC and also by crossing the blood–brain barrier (6, 69, 78). The microvascular endothelial cells in the CNS express the HCV receptors and allow the virus entry. Finally, the virus enters the CNS and allows the entry of cytokines and chemokines, which results in immune brain activation (79–81). The expression of CD-81, SR-BI, and claudin 1 on epithelial and endothelial cells takes place, but the entry of the virus can be prevented by developing antibodies derived from cerebral endothelium, and in this way, they can be used as entry targets for the virus in the brain (81, 82). The virus enters directly into the cells by invading the obstacles and altering the blood–brain barrier filter, as has been observed in HIV (83, 84). Based on the recent reports conducted using immune-histochemical and molecular techniques, it has been confirmed that HCV infects astrocytes and microglia and results in excess production of amino acids, which leads to neuronal death (83, 85, 86).

**Table 2:** Role of HCV in the development of neurological disorders.

S. No.	Neurological Disorders	Descriptions	References
1	**CNS disorders**	HCV induces many CNS disorders which includes cerebrovascular, neuropathology, encephalic inflammation, autoimmune, meningeal, and rheumatic disorders.	(65)
2	**Cryoglobulinemia**	In this case the circulating immunoglobulins resulting in organ damage.	(66)
3	**Cerebrovascular**	The role of HCV has been reported to be associated with may disorders. Flow of blood in the brain is affected by many factors.	(67)
4	**Inflammatory disorders**	In this case the body effects on its own immune system and the Rheumatoid arthritis are the best example of inflammatory disorders.	(68)
5	**PNS disorders**	HCV induces PNS disorders in 40-75% of the infected individuals. In this case, vessels become medium size.	(69)
6	**Neuropsychological disorders**	This includes a variety of disease disorders.	(69)
7	**Fatigue/fibromyalgia**	This is the results of associated chronic HCV infection. This results into muscle weakness.	(54)
8	**Cognitive impairment**	This is the condition in which patient’s cannot recognize any object easily and loss of memory and understanding.	(70)
9	**Depression**	HCV infection and the development of anxiety and depression with high frequency in the infected patients and affects life quality.	(28)
10	**Restless Legg syndrome**	The HCV infection also induces some restless in the leg during day and night.	(28)
11	**CAHD**	This is known as chronic acquired hepato-cerebral degeneration that includes the neurological disorders such as cognitive and behavioral changes in the HCV infected patients.	(38)
12	**PD- (Parkinson’s Disease)**	Parkinson’s disease is well known neurological disorders.	(71)
13	**Inflammation**	An inflammation plays an important role in the development of HCC.	(72)
14	**SICCA syndrome**	This is autoimmune disease and known to be associated with HCV and neurological disorders.	(73, 74)

## Evidence of HCV infection to CNS

The evidence of HCV infection to the CNS has been poorly understood because a very low level of viral RNA has been detected in postmortem brain tissue and cerebrospinal fluid (CSF) by the polymerase chain reaction (PCR) test, and that evidence does not strongly support the HCV infection and their replication because the breakdown of blood-borne barrier is possible after death (39). The detection of negative strands of HCV RNA by advanced molecular tools in CNS and brain tissue is the only confirmatory evidence that proves the virus replication and is considered a replicative intermediate and distinct viral quasi-species. It has been observed that the HCV-RNA was detected at a very low level (1,000–10,000-fold lower) in brain tissue as compared with liver tissue, which indicates that the brain tissue is not suitable for virus replication. The presence of unique sequences, including the internal ribosomal entry site (IRES), has been detected in the CNS, which is responsible for viral protein translation. Recently, the genetic compartmentalization of HCV in the CSF of HCV-infected patients with cognitively impaired symptoms has been demonstrated by deep sequencing techniques (87). Additionally, some molecular and immuno-histochemical techniques have proven that the microglia and astrocytes are the main targets for HCV infection in infected patients (3).

### Alteration of neurotransmission and metabolic activities

**Figure 2:**
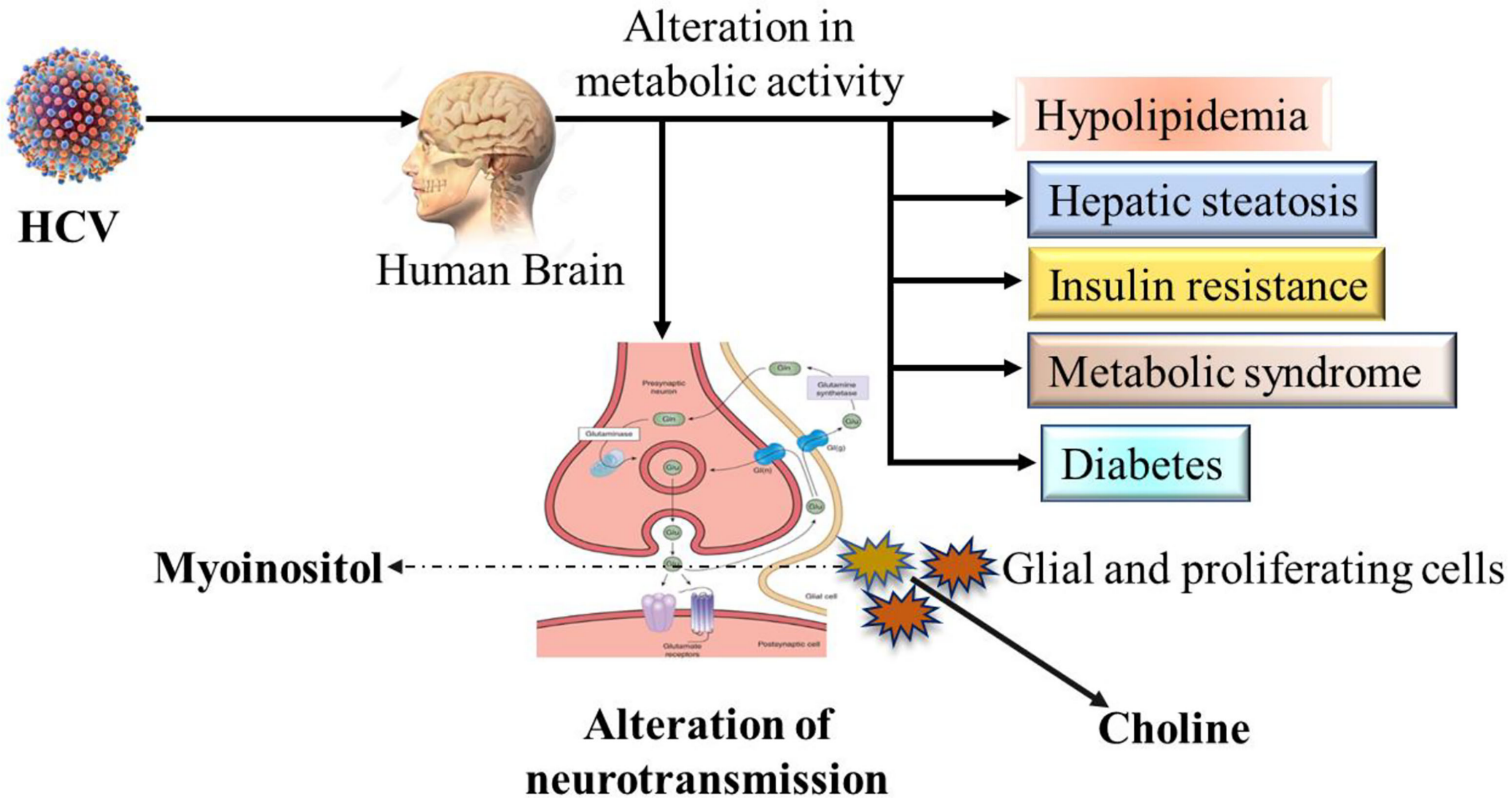
Possible schematic overview of neurotransmission alteration and affected metabolic activities by HCV infection in human brain.

Several metabolites are known as markers of neuroinflammation. The virus infection alters metabolic activity, which leads to an alteration of neurotransmission. The well-known metabolite marker choline is produced by glial and proliferating cells (**Figure 2**). The lower expression indicates the loss of neurons or dysfunction. Additionally, glial cells produce myoinositol and higher-level expression indicates gliosis (3, 65). The changes in serotonergic and dopaminergic neurotransmitter systems in the brain and basal ganglia have been demonstrated in 60% of HCV-infected patients by using photon emission computed tomography (SPECT) (88, 89). The high level of cytokines, including IL-1 and IL-6, interferes significantly with the neurotransmitter systems (3, 90). The neuroinflammation hypothesis has been confirmed by neuronal invasion and immune activation as well as increased perfusion in the basal ganglia (91). Finally, these studies show the abnormalities in the signaling and neuroinflammation and another inflammatory condition known as multiple sclerosis, is the result of chronic HCV infection (92).

### Cryoglobulinemia

The term cryoglobulinemia is defined as the presence of circulating immunoglobulins resulting in organ damage. These immunoglobulins can be re-dissolved at high temperatures. The causes of cryoglobulinemia are linked with HCV infections and autoimmune disorders (66). The two main types of cryoglobulinemia; type I cryoglobulinemia, consisting of vascular sludging and the second is type II and III cryoglobulinemia, which is the result of immune-mediated vasculitis due to overproduction of cryoglobulin by B lymphocytes (93). HCV infection results in the precipitation of immune complexes. This is reported in more than 70%–80% of cases associated with Type II immunoglobulin (Ig)M. The symptoms of cryoglobulinemia include leukocytoclastic vasculitis, transmural fibrinoid necrosis, peripheral neuropathy, and thrombotic lumen occlusion (94).

## Disorders of central nervous system (CNS)

### Cerebrovascular disease

The term “cerebrovascular disease” refers to a group of conditions in which the flow of blood in the brain is affected by many factors, such as the narrowing of blood vessels, the formation of clots, and blockage in the artery or rupture of blood vessels. HCV infection frequently results in acute and chronic cerebral vasculopathy. The level of viral RNA is directly correlated with the risk of cerebrovascular death in infected patients (67). Based on the data generated from retrospective and prospective studies, the clinical manifestation of HCV infection leads to many conditions such as lacunar syndrome, cardiovascular risk factors, and bleeding (67, 95). HCV infection induces a chronic systemic inflammatory state resulting in carotid plaque development and plaque instability, rupture, and thromboembolic events (96, 97). Additionally, chronic HCV infection also leads to imbalances in metabolic activities, especially the glucose homeostasis and lipid metabolism, as well as the induction of type II diabetes in the infected patients (69, 98). Additionally, ischemic events can cause non-cryo-bobulinemic vasculitis and systemic vasculitis (75, 99, 100).

### Inflammatory disorders

The inflammatory disorders are known as diseases caused by the result of an attack on the own immune system of the body. Rheumatoid arthritis is the best example of an inflammatory disease. The role and association of HCV infection in the inflammatory diseases of the CNS is well known. The development of leukoencephalitis results in multiple conditions including cognitive decline, tetraparesis, and aphasia, and the development of microglia nodules and progressive encephalomyelitis, psychomotor agitation, have been reported in literature (68, 101, 102).

## Peripheral nervous system disorders

Because of the infection by HCV, it has been reported that about 40%–75% of patients develop peripheral neuropathy and medium-sized vessels. This peripheral neuropathy has been observed in up to 60% of cryoglobulinemia patients and only 6% in CNS patients (69, 75, 103). Additionally, peripheral neuropathy has also been observed in patients without mixed cryoglobulinemia. The emergence of immune complexes is induced by viral infection and plays a significant role in the inflammation of vascular and perivascular systems (3, 104). Peripheral neuropathy frequently develops as symmetrical axonal sensory loss, which leads to leg weakness and loss of sensation in infected patients (105). The asymmetrical forms include the small fiber sensory polyneuropathy, which results in tingling and burning of the legs and feet. Sensation loss, paraesthesia, cramps, and numbness are the damage results of larger caliber fibers in advanced cases of neuropathy (106, 107). The neuropathy of non-contiguous nerves falls under mono or multiple neuropathies, and autonomic neuropathy is known as a rare form of neuropathy (75, 108, 109).

## Neuropsychological disorders

Neuropsychological disorders include a variety of disease and disorders. The disruption of the cerebral cortex and subcortical connections leads to the development of multiple neurological disorders with many clinical features. Apart from liver disease, HCV infection also triggers the development of neuropsychiatric disorders in approximately 50% of HCV-infected patients. These include fatigue, “brain fog,” cognitive deficits, as well as impaired life quality, which affect daily life activities. Chronic HCV-infected patients develop both psychiatric and cognitive impairments (69, 84, 86).

### Fatigue/fibromyalgia and cognitive impairment

Fatigue and cognitive impairment are the results of associated chronic HCV infection, which leads to a reduction in the life quality of infected patients. Fatigue differs from the central to the peripheral nervous system and is linked with muscle weakness. The peripheral fatigue is produced by changes at distal to the neuromuscular junction while the central fatigue originates at CNS results into muscle weekness. The factors, including psychological, social, and physical ones that play an important role in the development of fatigue (3). Approximately 50% of infected patients develop fatigue, which indicates chronic HCV infection. Fatigue can develop at anytime and there is no relationship between viral RNA and its genotype as well as histology of the liver in the development of fatigue (110). The loss of energy and chronic fatigue with pain are the main symptoms of chronic HCV infection. The abnormal circulation of thyroid-stimulating hormones may also result in a high frequency of fatigue (110). The HCV-associated cognitive impairment and neuropsychiatric symptoms include depression, attention problems, reduction of learning and reasoning ability, reduction of recall, impairment of serotonin transmission concentration, depression, loss of memory, ability to work, make decisions and judgments, which have been reported in the literature (70, 111). Additionally, muscle and joint pain and sleep disturbances are also reported in HCV-infected patients. Based on the current research, it has been hypothesized that the proper cure for HCV infection can reduce the development of fatigue (75, 112). The association between HCV infection and the triggering of fatigue and hepatic fibrosis has already been reported (113). The reduction of dopamine in the limbic system results in cytokine-induced reduction of tetrahydrobiopterin, and the association between fatigue and peripheral inflammation has been discussed in many research papers (114–117). In a recent study, it was observed that 19% of HCV-infected patients fulfilled the diagnostic criteria of fibromyalgia (77). Additionally, arthralgia is frequently observed in cryoglobulinemia vascuarthritis patients (77, 118–120).

### Depression

A literature review has discussed the association between HCV infection and the development of anxiety and depression with high frequency in infected patients and affects quality of life (3). Patients with psychiatric disorders have more HCV infections than the general population, and it has been observed to the HCV genotype 3 can affect 20%–50% of individuals (3, 69, 121, 122). This association significantly affects the prognosis and antiviral therapy in those patients (123). Therefore, there is an urgent need to use a multidisciplinary approach to ensure the management and treatment of depression (115). The onset of depression may also be observed using intravenous substances, fatigue, and cognitive decline (124, 125). The endotoxins and peripheral cytokines also play a significant role in the development of depression in HCV-infected patients. These cytokines can inhibit neurotransmission and induce metabolic enzyme activity (126, 127). Additionally, HCV infection may also induce the depression with the secondary factors such as diabetes, insulin resistance, metabolic syndrome, heart disease, and inflammatory conditions like joint pain, arthritis, motor, and sensory neuropathies, serotonin deficiency, a corticotropin-releasing hormone which leads to more release of inhibitory neurotransmitters and finally results in the development of depression (65, 115, 128, 129).

### Restless legs syndrome

Apart from cognitive manifestations, motor-neuron problems may also arise in chronic HCV-infected patients. The infected patients feel the movement of leg during evening and night. This syndrome may put the infected patients at greater risk. The use of interferon-alpha as therapy and cirrhosis are particularly associated with the RLS (130, 131).

## Chronic acquired hepato-cerebral degeneration

CAHD is a neurological disorder of HCV-infected patients who develop movement syndromes as well as cognitive\behavioral changes. The symptoms are not fully understood and are poorly described in medical therapy. The development of CAHD is believed that due to the accumulation of ammonia or manganese in the brain, which leads to neurodegeneration, but the mechanism of pathogenesis is not yet clear (132, 133). Meige’s syndrome is an unusual condition of dystonia in which patients face problems like a spasm in the muscle of the eye, jaw, tongue, and facial muscles due to nervous system and neurodegenerative disorders. In a recent study, Meige’s syndrome and cognitive–behavioral conditions were discussed with liver cirrhosis and hepatocarcinoma 60-year-old patients and the diagnosis of CAHD was confirmed, and indicating that this syndrome may be healed by the transplantation therapy (134).

## Parkinson’s disease

PD is a long-term progressive neurodegenerative disorder of the central nervous system that mainly affects the motor system, resulting in the complex problem of balancing, movement, shaking, and walking difficulty due to loss of neurons in the substantia nigra (SN) (71, 129, 135, 136). Currently, no therapy is available, but many treatment options are suggested to resolve this disorder. This is the second most common disorder, reported in 10–18 per 100,000 individuals every year. The association of HCV infection and interferon (IFN) therapy in the induction of neurodegenerative disorders that lead to PD has been reported recently in some observational studies (71). Additionally, there are many etiological factors, including genetic and environmental conditions, that trigger the process of neurological disorders (137). The independent risk factors that have also been recognized that include viral infections such as influenza, Coxsackie, Japanese encephalitis, HIV, and HCV associated with PD (129).

### Possible mechanisms of HCV-PD association

**Figure 3:**
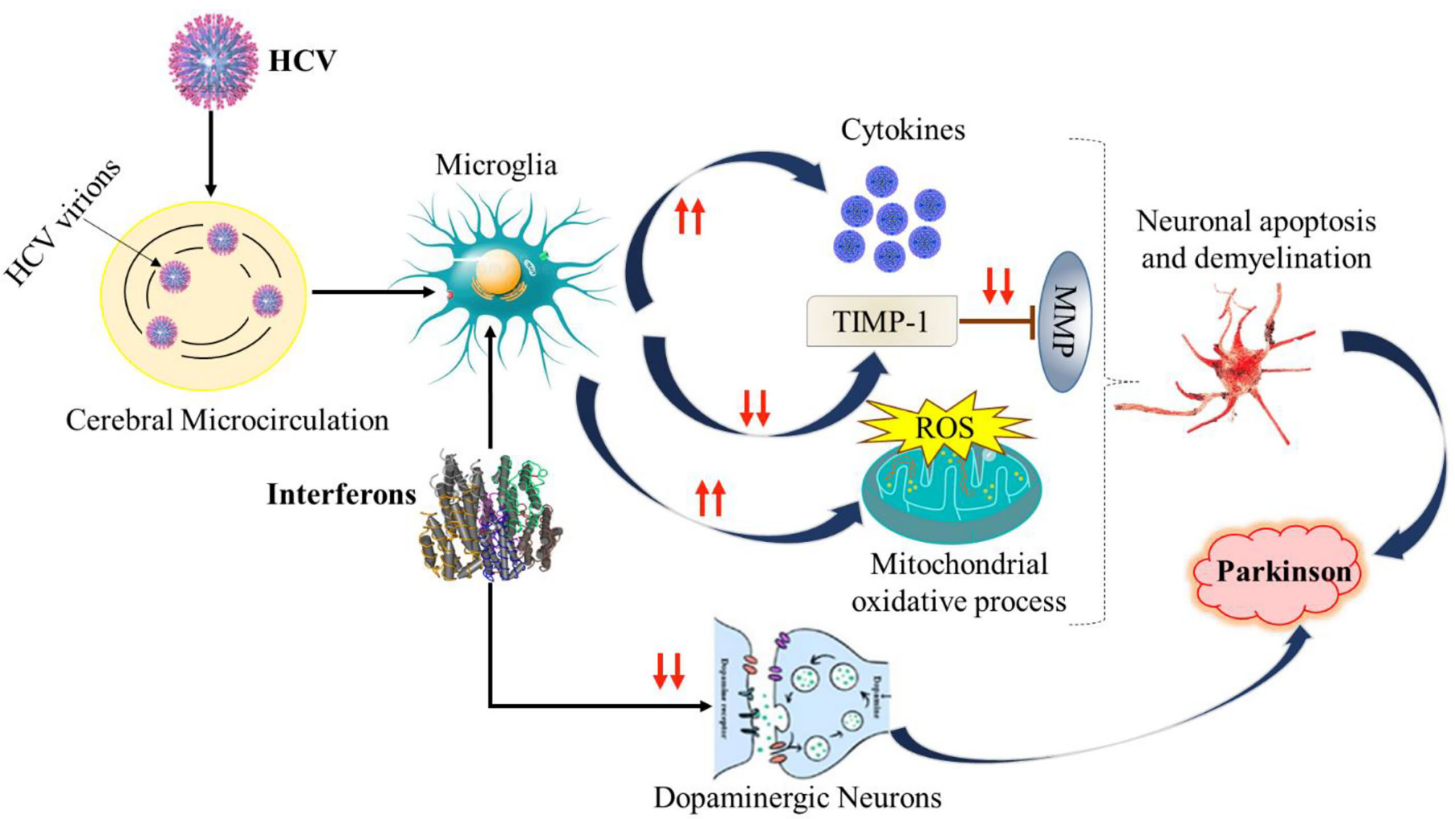
Depiction of Possible mechanisms of HCV-PD. TIMP-1, Tissue inhibitor of metalloprotease-1; MMP, Matrix metalloproteinase. ROS, Reactive oxygen species.

The mechanism of PD and the association of HCV has been poorly understood due to a lack of data and the accuracy of detection methods for viral RNA that confirm the viral replication in the CNS. The viral RNA was identified in post-mortem brain tissue. The role of macrophages in the CNS has been highlighted because the viral was amplified more from lymphoid tissues as well as the serum compared to brain tissue (138). In a study conducted the HCV virions were identified using two different approaches including anti-NS3 monoclonal antibodies HCV receptors on the brain microvasculature, which indicates the virus infection to brain cells (79). In another study, it was shown that viruses can induce microglial damage to brain cells. The infected cells release neurotoxins and cytokines that affect the metabolism and function of brain cells (139, 140). Additionally, it was observed that HCV can reduce the expression of TIMP-1, which is responsible for neuroprotection, by inhibiting the matrix metalloproteinase (MMP) enzymes (**Figure 3**). It has already been reported that HCV infects microglia/brain macrophages and enters the brain and induces the inflammation that leads to neurodegeneration. The other mechanism hypothesized, based on an observational study by Wu et al. in Taiwan, is that the association of HCV-PD induces the striatal dopaminergic neurotransmission changes or metabolic syndrome and the induction of insulin resistance (129, 141).

### Inflammation in PD

Inflammation plays an important role in the pathophysiological pathway of PD and disease progression (72). A significant discovery about the HLA complex has been made and the genome-wide association analysis has revealed that higher expression of HLA-coding genes, as well as some reduction of other HLA types. Similar results were also found in inflammatory bowel disease (IBD) and Crohn’s disease (142). The animal models have provided vital information about the evaluation of various compounds against inflammation in the CNS (143). Some of the animal models showed the activation of microglia and upregulation of pro-inflammatory cytokines in postmortem PD brains. During the last few years, significant efforts have been made to access the inflammatory factors either in the peripheral blood or the CSF of PD individuals (144). Recent studies show that there are many genes that have a strong association with PD and the immune response of microglia and astrocytes of the CNS. This group of genes are known as the α-synuclein gene (SNCA), leucine-rich repeat kinase 2 (LRRK2) gene, PTEN-induced putative kinase 1 (PINK1) gene, Parkin, and the DJ-1 gene (72, 145). Additionally, DJ-1 has also been identified to be associated with PD and inflammation. This gene is partially expressed in microglia and astrocytes. A total of 17 genes have been identified to be associated with inflammation and familial transmission of PD. The PARK6, PINK1, and PARK7 mitigate the inflammatory response to mitochondria-released DAMPs (146, 147). In addition to the role of microglia and astrocytes in inflammation and PD, peripheral inflammation and PD-associated genes also play a significant role in the chronic inflammation and progression of neurological disorders (72, 147, 148).

## Hepatitis c virus and SICCA syndrome

SICCA syndrome is an autoimmune disease that is also known as Sjogren syndrome. The term SICCA refers to eye and mouth dryness. The symptoms include dry eyes and mouth as well as other connective tissues such as rheumatoid arthritis. This is an inflammatory disease of the glands and other tissues in the body that produce tears and saliva. This is most common in women. Approximately 90% of patients with this syndrome are women of mid or older age. The prevalence of SICCA symptoms was observed in 10%–30% of HCV patients (73). The presence of anti-SSA or anti-SSB antibodies and histology of salivary glands confirms the positive association of HCV in the infected patients (74, 77).

## Conclusions and future perspectives

Based on the latest findings and published reports and fast-moving technological advancements, including omic studies, have provided more detailed and profound information about the role of HCV in cancer and neurological disorders. During the last few decades, the treatment of viral hepatitis has gone through major advancements with effective therapy. But, despite this, HCV infection is the main cause of liver diseases and HCC that lead to high mortality. DAAs therapy has been observed to be the most revolutionized antiviral therapy that will reduce the HCV infection, liver diseases and mortality, but still, more detailed studies are urgently required to eradicate the HCV and long-term protection of neurologically impaired patients. The risk of HCC persists even after the treatment in cirrhotic individuals because of other non-viral factors such as obesity, alcohol use, non-alcoholic fatty liver disease (NAFLD), non-alcoholic steatohepatitis (NASH), and other autoimmune diseases. There are many reports that discuss the significant reduction in the success rate of DAAs in HCV eradication for those patients who have co-existing active HCC, which leads to tumor-driven immunosuppression. Targeting the immunosuppressive patients, host-signal targeting approach and including the antitumor responses by cell dysfunction will also be highly fruitful to combat the HCV-induced HCC. The multi-omics single-cell data and their analysis will provide an opportunity to identify the nucleic acid inflammatory pathways in each hepatic cell population and can be used for drug discovery and biomarkers against HCV. Additionally, with the proper diagnosis of HCV, recommending proper medication, counseling, and care as well as managing extrahepatic complications of the infected individuals will be more appropriate for the disease management. In this review, we have significantly summarized the most updated information about the molecular basis of HCC induced by HCV as well as CNS impairment and disorders in HCV patients. The currently available DAAs for viral clearance and elimination do not significantly protect drug users. There is an urgent need to know and understand more about the complex microenvironment of the liver and viral infection as well as cirrhosis. The prevention of HCV infection and transmission by protective vaccinations is urgently needed to protect the global population as well as to reduce the occurrence of neurological disorders.

## Author contributions

MS: conceptualization, original draft preparation, and writing. SS: Supervision, conceptualization; writing original draft, reviewing, and editing. MK: reviewing and editing. EA: Reviewing and editing. All authors listed have made a substantial, direct, and intellectual contribution to the work and approved it for publication.

## Funding

The authors extend their appreciation to the Deputyship for Research & Innovation, Ministry of Education in Saudi Arabia for funding this research work through project number IFPRP:142-141-1442, and King Abdulaziz University, DSR, Jeddah, Saudi Arabia.

## Conflict of Interest

The authors declare that the research was conducted in the absence of any commercial or financial relationships that could be construed as a potential conflict of interest.

## Publisher’s note

All claims expressed in this article are solely those of the authors and do not necessarily represent those of their affiliated organizations, or those of the publisher, the editors and the reviewers. Any product that may be evaluated in this article, or claim that may be made by its manufacturer, is not guaranteed or endorsed by the publisher.

## Abbreviations

CDKN1A, cyclin-dependent kinase inhibitor 1A; CAP, controlled attenuation parameter; CAHD, chronic acquired hepato-cerebral degeneration; CNS, central nervous system; CSF, cerebrospinal fluid; DAAs, direct-acting antivirals; DC, dendritic cells; EHMs, extra hepatic manifestations; EMT, epithelial-mesenchymal transition HATs, histone acetyltransferases; HCV, hepatitis C virus; HCC, hepatocellular carcinoma; HDACs, histone deacetylases; IRES, internal ribosomal entry site; MMP, matrix metalloproteinase; ncRNAs, non-coding RNAs; Nk, natural killer; ORF, open reading frame; PBMC, peripheral blood mononuclear cells; PCR, polymerase chain reaction; PD, parkinson’s disease; PTEN, phosphatase and tensin homolog; RLS, restless legs syndrome; ROS, reactive oxygen species; SVR, sustained virological response; SFRP1, secreted frizzled-related protein 1; TGF-β, transforming growth factor-beta; Toll-like receptors (TLRs),VCTE, vibration-controlled transient elastography.
